# Computational Study of Unfolding and Regulation Mechanism of preQ_1_ Riboswitches

**DOI:** 10.1371/journal.pone.0045239

**Published:** 2012-09-17

**Authors:** Zhou Gong, Yunjie Zhao, Changjun Chen, Yi Xiao

**Affiliations:** Biomolecular Physics and Modeling Group, Department of Physics, Huazhong University of Science and Technology, Wuhan, Hubei, China; University of South Florida College of Medicine, United States of America

## Abstract

Riboswitches are novel RNA regulatory elements. Each riboswitch molecule consists of two domains: aptamer and express platform. The three-dimensional (3D) structure of the aptamer domain, depending on ligand binding or not, controls that of the express platform, which then switches on or off transcriptional or translational process. Here we study the two types of preQ_1_ riboswitch aptamers from *T. Tengcongensis* (denoted as Tte preQ_1_ riboswitch for short below) and *Bacillus subtilis* (denoted as Bsu preQ_1_ riboswitch for short below), respectively. The free-state 3D structure of the Tte preQ_1_ riboswitch is the same as its bound state but the Bsu preQ_1_ riboswitch is not. Therefore, it is very interesting to investigate how these riboswitches realize their different regulation functions. We simulated the unfolding of these two aptamers through all-atom molecular dynamic simulation and found that they have similar unfolding or folding pathways and ligand-binding processes. The main difference between them is the folding intermediate states. The similarity and difference of their unfolding or folding dynamics may suggest their similar regulation mechanisms and account for their different functions, respectively. These results are also useful to understand the regulation mechanism of other riboswitches with free-state 3D structures similar to their bound states.

## Introduction

Riboswitches are RNA regulatory elements that perform regulation functions through conformational switch between ligand-free and ligand-bound states [1]–[3]. Therefore, the three-dimensional (3D) conformations of free and bound states of riboswitches are crucial to understand their regulation function and mechanism. Up to now, the 3D structures of the ligand-bound states of many riboswitch aptamers have already been solved. Recently the ligand-free 3D structures of some aptamers have also been solved [4]–[6]. However, it is found that nearly all of these free-state 3D structures adopt bound-like structures [7], [8]. This proposes a question: How do these riboswitches perform regulation functions in free form?

**Figure 1:**
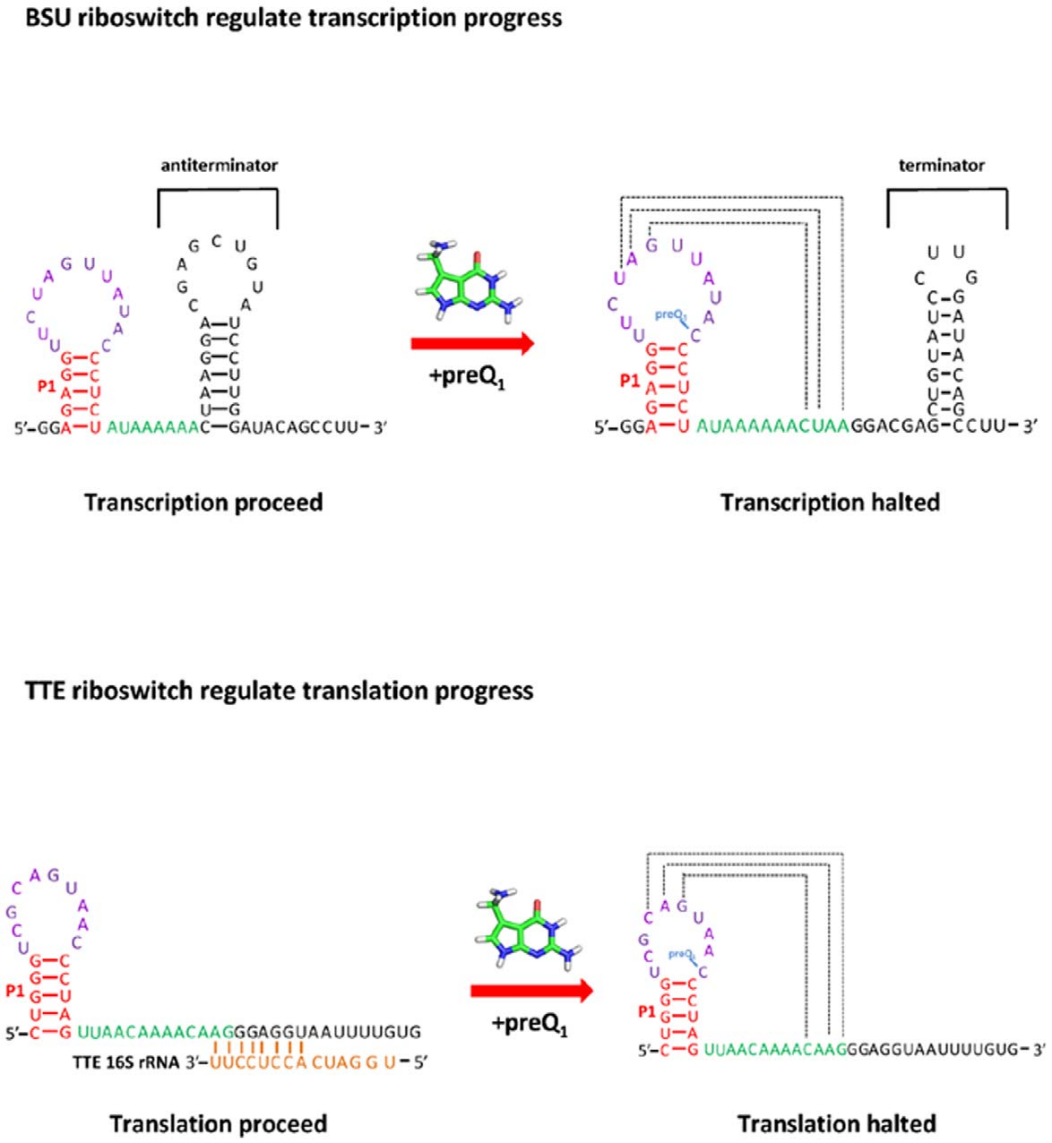
Regulation functions for two types of preQ_1_ riboswitches.

**Figure 2:**
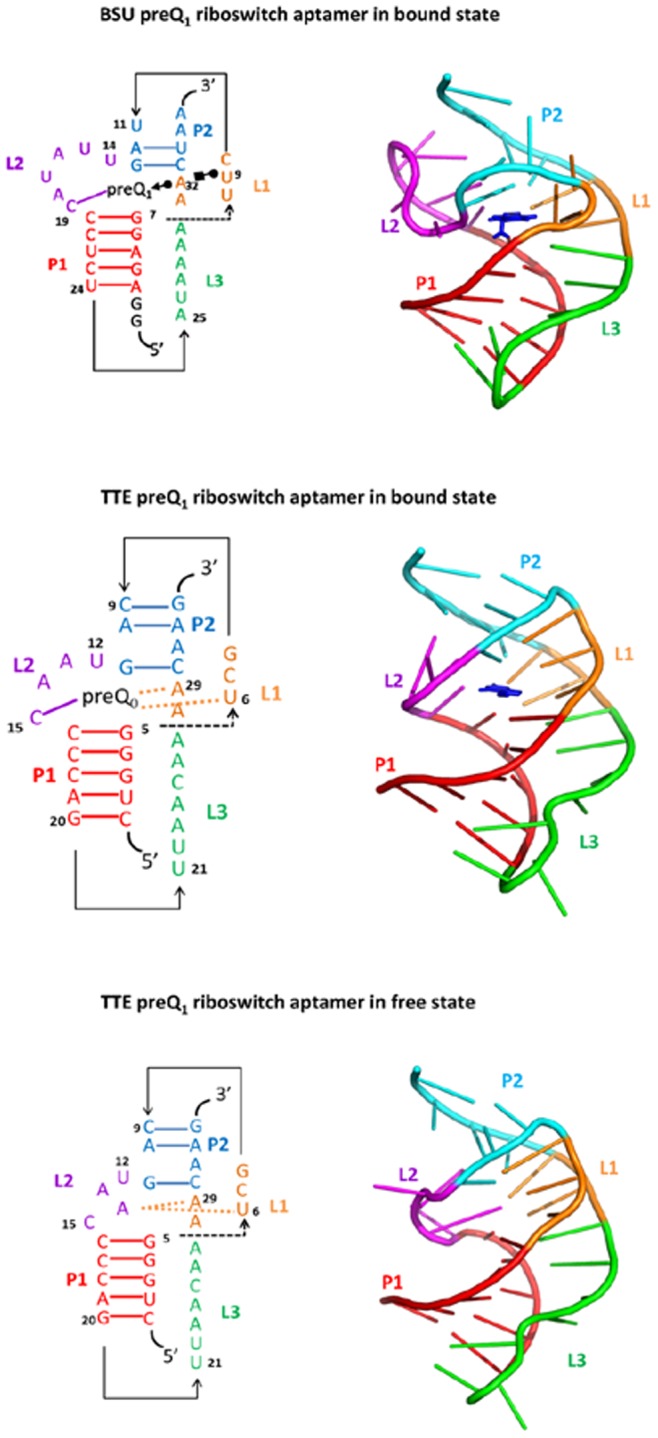
Secondary and tertiary structures of two types of the preQ_1_ riboswitch aptamer domains. The bound preQ_1_ is depicted by a licorice representation. The different parts (P1, P2, L1, L2, and L3) are color-coded.

Recently, a series of experiments have focused on two types of preQ_1_ riboswitches. One of them is from *Bacillus subtilis* [9], [10]. The other one comes from *T. Tengcongensis* [11]. These two types of riboswitches have different functions (Figure 1). The former regulates transcription while the latter translation. In ligand-bound state, the aptamers of both preQ_1_ riboswitches adopt similar H-type pseudoknot structure. While in ligand-free state, Tte preQ_1_ riboswitch aptamers has almost the same 3D structure as its ligand-bound state [11] (see Figure 2). Though the structure of Bsu preQ_1_ riboswitch aptamer in free state is not available, the free state structure is thought to be different as their bound structure [9], [12]. The regulation of the Bsu preQ_1_ riboswitch was considered to be kinetically controlled [9]. Jenkins et al [11] suggested that Tte preQ_1_ riboswitch would have a different regulation mechanism. In this paper we show that the two preQ_1_ riboswitches may use similar physical mechanism to control regulation processes.

The kinetic control mechanism depends on folding pathways of the aptamers. At present micro to millisecond timescale simulations of RNA folding using conventional all-atom molecular dynamics (MD) methods are still very difficult. Some simplifications are needed. For example, Feng et al. [13] used an all-atom Gō-model to investigate the folding mechanism of the Bsu preQ_1_ riboswitch aptamer. They found that the preQ_1_ riboswitch aptamer may fold along a directional pathway: the P1 stem-loop folds first, then the A-tract region establishes tertiary contacts with the P1 stem, and finally the pseudoknot P2 forms, i.e., the folding is along a single route from the 5′ to 3′ end and in the same direction as the transcription proceeds. However, as pointed by the authors [13], the Gō-model smoothed out the folding free energy landscape and cannot localize the possible intermediate states with non-native interactions.

Another MD approach to study RNA folding is high-temperature unfolding simulation, which has served as a powerful tool for investigating dynamics involving large conformational changes on computationally tractable timescales [14]–[16]. Previous studies suggested that the unfolding events for proteins could reflect the main attributes of the folding progress [17]–[19]. Recent studies of RNA hairpins have also shown that construction of folding pathways, including transition state ensembles, is possible using high temperature unfolding [20]. Csaszar et al. revealed early unfolding events of RNA using all-atom molecular dynamics simulation on elevated temperature 400 K [21]. Zhang et al. found the multiple unfolding pathways, diverse transition states, and various intermediate structures in the unfolding simulation of a pseudoknot within gene 32 mRNA of bacteriophage T2 [22]. Here we will perform a series of unfolding simulations on high-temperature to investigate the folding/unfolding pathways and mechanisms of preQ1 riboswitch.

## Results and Discussion

**Figure 3:**
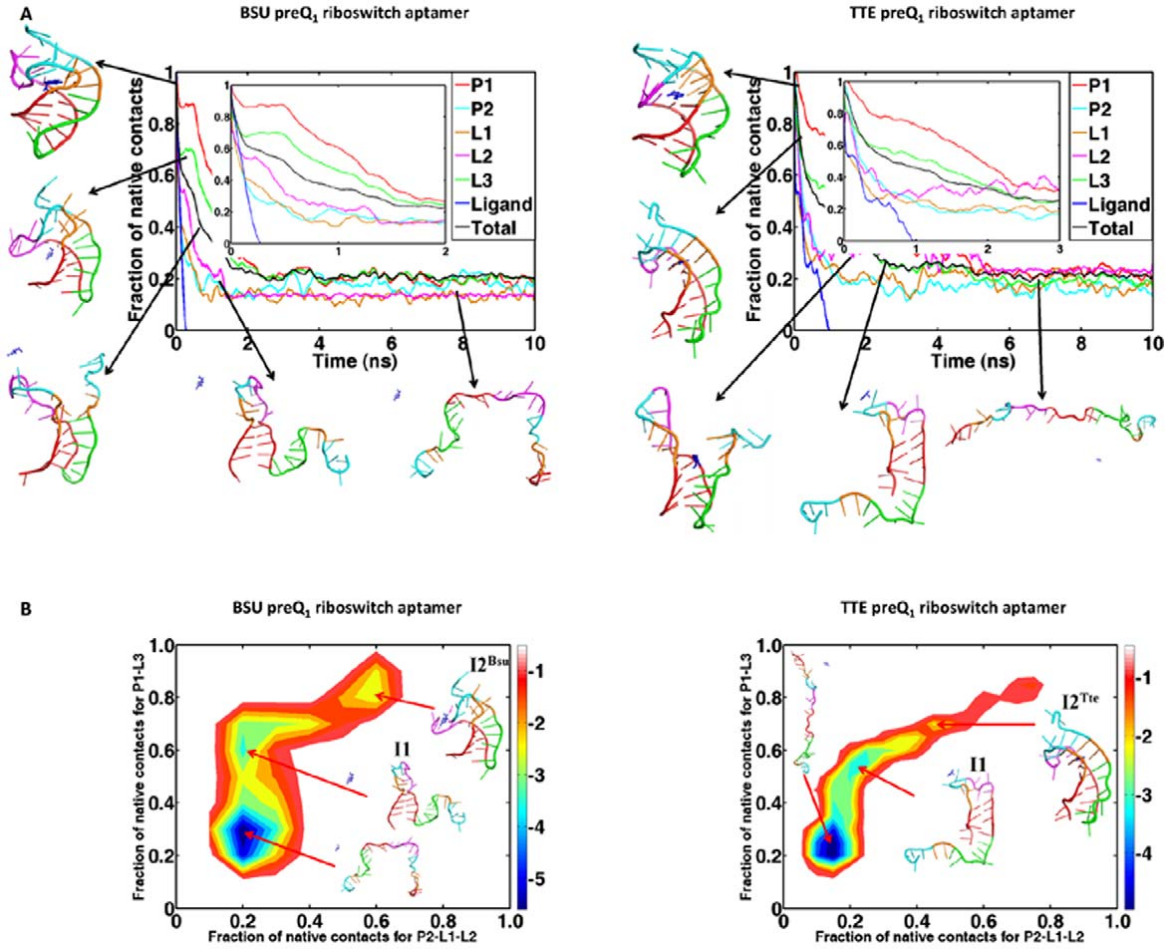
Unfolding pathways and free energy landscapes of Bsu and Tte preQ_1_ riboswitch aptamers in presence of the ligands at 400 K: (A) the time evolution of the mean fraction of native contacts in the aptamer during the unfolding simulations. The different curves describe different parts; (B) the two-dimensional unfolding free energy landscapes. The order parameters are the fractions of native contacts for P1-L3 and P2-L1-L2, respectively. The surrounding structures are representatives of the conformations observed during the unfolding processes.

Figure 3A describes the time evolution of the mean fraction of native contacts (NC) in Bsu and Tte preQ_1_ riboswitch aptamers as well as their components over the ten simulated unfolding trajectories at 400 K in the presence of ligands. It shows that the unfolding processes of both aptamers are similar: the P2 helix first loses its native contacts, then the A-tract L3 loses its tertiary contacts with P1 region and finally the P1 stem unfolds. This is essentially along a single route from the 3′ to the 5′ end, just the reverse direction of transcription and the folding pathway of the Bsu aptamer suggested by Feng et al [13].

Figure 3B is the two-dimensional unfolding free energy landscapes of the two aptamers built from the ten simulated trajectories in the presence of ligands, respectively. The order parameters are fractions of native contacts in the triplex P1-L3 and binding pocket region adding pseudoknot part P2-L1-L2. These free-energy landscapes further confirm the hierarchical unfolding pathways of the two aptamers. Furthermore, both free-energy landscapes show two intermediate states: one has a flexible structure but with stable hairpin P1 (denoted as I1) and another is different for the two aptamers: for Bsu aptamer the intermediate state (denoted as I2^Bsu^) has a structure with opening P2 and binding pocket but stable triplex P1-L3; on the other hand, the intermediate state of Tte aptamer (denoted as I2^Tte^) has a structure that is very close to the ligand-bound one but the P2 helix only has one of the base pairs G11:C30 remained and the base pairs C9:G33 has broken (see discussions in the following).

**Figure 4:**
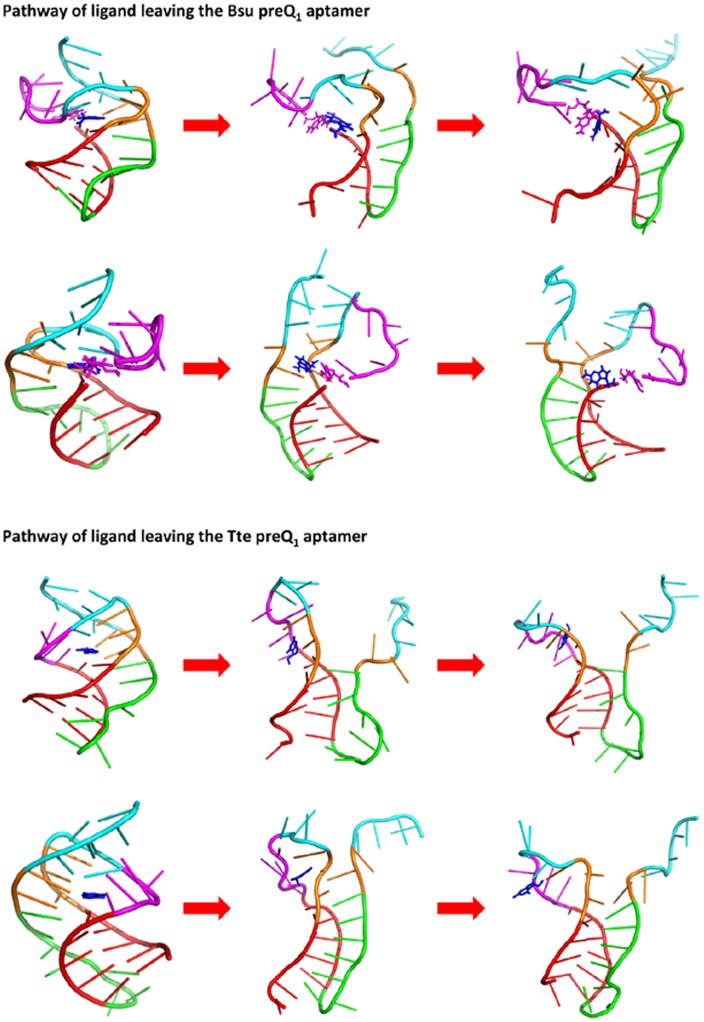
Two pathways for the ligand preQ_1_ move away from the aptamers. Top: the major groove side of P1; Bottom: the minor groove side of P1.

It is also shown on Figure 3A that the release of ligand occurs at the early stage of unfolding progress during the P2 helix breaks and the binding pocket (L1 and L2 loops) opens but before the P1-L3 triplex unfolds. The pathways that the ligands released from the two aptamers are also similar and along two directions: from the major or minor groove of P1 (Figure 4). This suggests that the ligand binds to the aptamer in the later stage of the folding process, especially after the formation of the P1-L3 triplex. The very recent experimental result described such a picture of ligand binding to the preQ_1_ aptamer domain [23]. Other experimental results also suggested that the P1 hairpin might already form before the ligand binding [9], [10].

**Figure 5:**
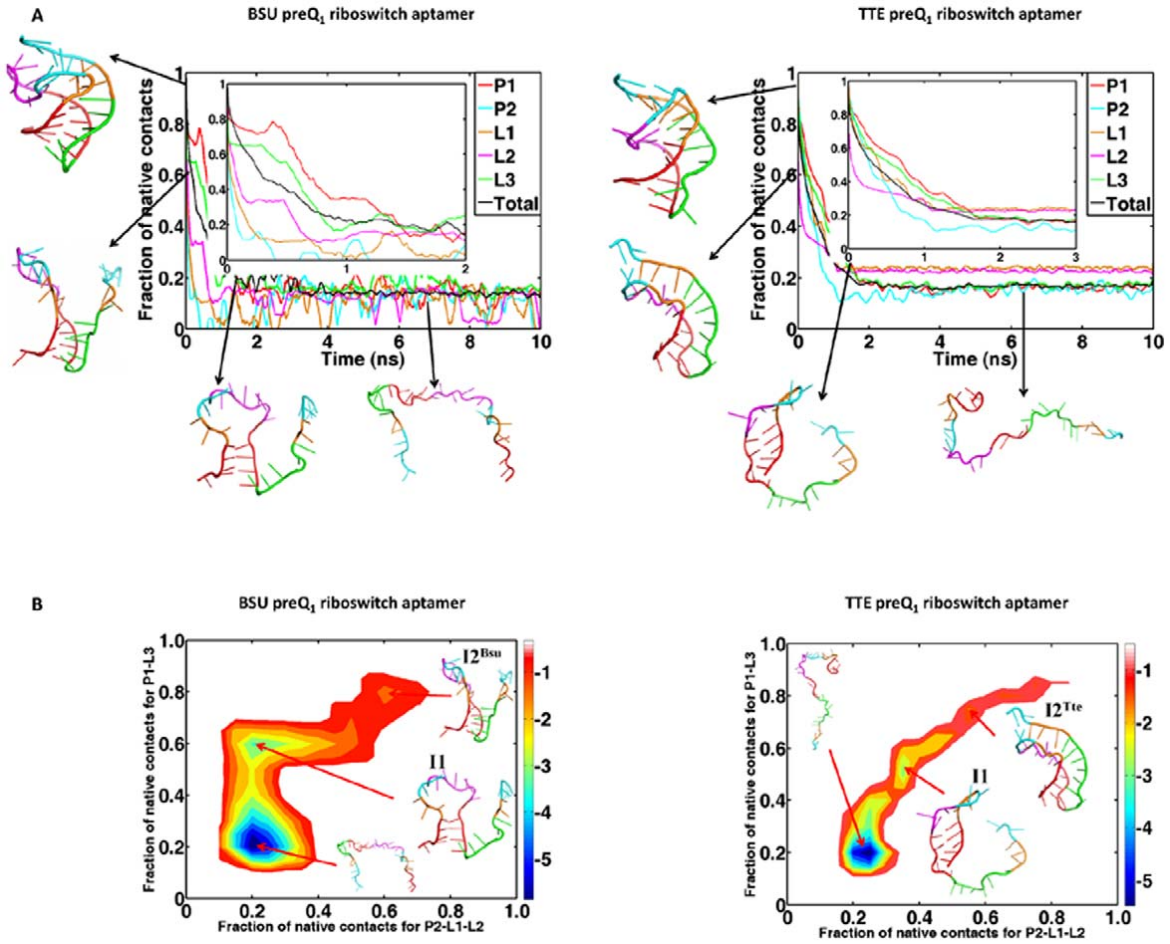
Unfolding pathways of the two preQ_1_ riboswitch aptamers at 400 K in absence of the ligands: (A) the time evolution of the mean fraction of native contacts in the aptamer during the unfolding simulations. The different curves describe different segments; (B) the two-dimensional unfolding free energy landscapes. The order parameters are the fractions of native contacts for P1-L3 and P2-L1-L2, respectively. The surrounding structures are representatives of the conformations observed during the unfolding process.

In the absence of ligands, we find that the global behaviors of the unfolding process of the two aptamers are also similar with each other as well as those in the presence of ligands (Figure 5): the P2 helix first loses its native contacts, then the A-tract L3 loses its tertiary contacts with P1 region and finally the P1 stem unfolds. The two-dimensional free energy landscapes of the Bsu and Tte aptamers (Figure 5B) are also similar to those in the presence of ligands. They also have two intermediate states although their stabilities have some changes in comparison with those in the presence of ligands. For the Bsu preQ_1_ riboswitch aptamer, the intermediate state I1 is still very stable and the intermediate state I2^Bsu^ becomes less stable now. This implies that the ligand may have certain effect on the formation of the triplex P1-L3 but not on the folding of the hairpin P1. However, it should be pointed out that the intermediate state I2^Bsu^ is very stable at 300 K in the absence of ligand (see supplementary Figures S1 and S2). For the Tte preQ_1_ riboswitch aptamer, the two intermediate states and their stabilities are very similar to those in the presence of ligand, i.e., the stability of the intermediate state I2^Tte^ has not be reduced significantly due to the absence of the ligand. This is different from the Bsu preQ_1_ riboswitch aptamer.

Since 400 K is higher than the transition temperatures of both aptamers, their starting structures are unstable and unfold quickly. So the two-dimensional free energy landscapes at 400 K cannot reflect the stability of the starting structures at the normal temperature. We also performed simulations at 300 K and the results are presented in the supplementary Figures S1 and S2. At 300 K the ligand-bound experimental structure of Tte preQ_1_ riboswitch aptamer is very stable but the ligand-free experimental structure is unstable and the more stable structure is just I2^Tte^. This can also be seen clearly from Supplementary Figure S3, which shows the variations of the distances between the nucleotides of the two base pairs in P2 region during the simulations. For the Bsu preQ_1_ riboswitch aptamer, the situation is similar: the ligand-bound experimental structure is stable, although one of the base pairs in P2 has broken, while the ligand-free experimental structure is not stable and the more stable structure is I2^Bsu^. These indicate that in the presence of ligands the stable structure of the two aptamers at the normal temperature are their experimental structures while in the absence of ligands their stable structure at the normal temperature are I2^Bsu^ and I2^Tte^, respectively. In I2^Bsu^ all the base pairs in P2 have broken while in I2^Tte^ only one of the base pairs in P2 was broken (see Supplementary Figures S3 and S4).

The results above show that the two aptamers have similar unfolding pathways no matter the ligands present or not. The main difference between them is that for Tte preQ_1_ riboswitch aptamer the structure of the intermediate state I2^Tte^ can keep part (one base pair G11:C30) of P2 helix but the Bsu preQ_1_ riboswitch aptamer cannot. Such different unfolding behaviors of these two aptamers come from different conformational features of them. In Tte preQ_1_ riboswitch aptamer all the nucleotides in pseudoknot part have formed stable base-paring or non-nearest-neighbor stacking interactions (bases from different sides of pseudoknot stack with each other), especially the C9 and G33 form the strongest G:C Watson-Crick base pair at the end of the pseudoknot, which make terminal of riboswitch remain stable. By contrast, there are no non-nearest-neighbor base-stacking interactions between two chains of the pseudoknot part in Bsu preQ_1_ riboswitch aptamer, in addition to unpaired terminal base. These different structural features make the Tte preQ_1_ riboswitch aptamer have more stable pseudoknot part than Bsu preQ_1_ riboswitch aptamer.

There is one more factor that makes the binding pocket and P2 of the Tte preQ_1_ riboswitch aptamer more stable in the ligand free form. In this case, the nucleotide A14 in L2 region inserts into the binding pocket and forms hydrogen bonds with A29 instead of the ligand preQ_1_, which makes the binding pocket keep stable and holds the tail of the aptamer to form pseudoknot shape. This may explain why part of the pseudoknot structure can still maintain in ligand-free state. By contrast, the L2 region of Bsu preQ_1_ riboswitch is 6-nt long, which has two more nucleotides than that in Tte preQ_1_ riboswitch aptamer. The extra-long length of the L2 region increases its flexibility and makes the nucleotides in L2 region difficult to turn back into the binding pocket to form effective interactions with the tail part in the absence of the ligand.

Such similarity and difference between the two aptamers in unfolding or folding behaviors may be related to their different functions. In the presence of ligands both Bsu and Tte preQ_1_ riboswitch aptamers form pseudoknot structures due to ligand binding and make their express platforms switch to “OFF” conformations to stop transcription or translation (Figure 1). For Bsu preQ_1_ riboswitch aptamer, the formation of the pseudoknot structure makes the express platform form a terminator that halts transcription. For Tte preQ_1_ riboswitch aptamer, the formation of the pseudoknot structure makes the two 5′-end nucleotides AG of ribosome binding sequence closed in P2 helix and avoid exposure to ribosome. In the absence of the ligands, both Bsu and Tte preQ_1_ riboswitch aptamers should form structures that make their express platforms switch to “ON” conformations to allow transcription or translation to proceed. For Bsu preQ_1_ riboswitch aptamer, it needs forming an anti-terminator and this requires the four nucleotides CUAA at the 3′ end of the aptamer do not form pseudoknot base pairs with the hairpin P1 as in the ligand-bound structure (Figure 1). This just corresponds to the stable state I2^Bsu^ at the normal temperature in the absence of the ligand. This may be why the Bsu preQ_1_ riboswitch aptamer needs a long L2 loop that makes it difficult to form interactions with the tail part and forbid the formation of the pseudoknot in the absent of ligand. For Tte preQ_1_ riboswitch aptamer, in order to allow the translation to proceed, it only needs the two 5′-end nucleotides AG of ribosome binding sequence apart from the hairpin P1 and the nucleotide C30 can still form pseudoknot base pair (Figure 1). This is just the stable state I2^Tte^ at the normal temperature in the absence of the ligand. This indicates that the kinetic behaviors of the two aptamers can satisfy the requirement of their functions.

## Methods

### Structural Analysis

The preQ_1_ riboswitch aptamer domain adopts an H-type pseudoknot with two stems (P1 and P2) and three loops (L1, L2 and L3) in the ligand-bound state. Figure 2 plot the secondary structure and tertiary structure of preQ_1_ riboswitch. The overall structures of these two riboswitches are very similar, with backbone RMSD of 4.0 Å. It shows that the stem P2 stacks over the P1 and L1 and the ligand preQ_1_ locates in a closed binding pocket around by several nucleotides from different part of aptamer. Previous studies [9], [10] showed that in the Bsu preQ_1_ riboswitch the P2 helix forms only when the ligand preQ_1_ binds to the aptamer while in the Tte preQ_1_ riboswitch the pseudoknot P2 can form in both ligand-bound and ligand-free states and they adopts the similar H-type pseudoknot [11]. Furthermore, in Bsu riboswitch the adenine-tract (A-tract) L3 of the tail form a stretch of “A-amino kissing motif” [24] contacting in the minor groove of the P1 stem and the L2 loop is unusual 6-nt long which lies in the minor groove of P2 [9], while in the Tte preQ_1_ riboswitch the L3 region has much less contacts with P1 and the L2 loop have only 4-nt long.

### Simulations Strategy

All minimization and MD simulations are carried out using the sander program in AMBER 11 software package [25]. Previous studies have shown that the Amber ff98 as well as modified ff99 force fields can stabilize the RNA structures during the GB and explicit solvation simulations [26]–[30]. Here, we choose the appropriate Amber ff98 force field and HCT model (Tsui-Case parameters) [31] GB/SA implicit solvation model for the simulations. We simulated two sets of unfolding trajectories of the Bsu preQ_1_ riboswitch aptamer starting from the NMR structure (the first structure of PDB 2L1V) with and without the ligand preQ_1_, respectively. For the Tte preQ_1_ riboswitch, we also simulated two sets of unfolding trajectories starting from the bound-state (PDB ID: 3Q50) and free-state (PDB ID: 3Q51) experimental structures, respectively. All of the structures are simulated at the temperatures 300 K and 400 K. The canonical (constant T) ensemble is chosen for the simulation and the Langevin thermostat is used to control the temperature using a collision frequency of 1.0/ps [32]. The effective ion concentration is 0.2 M, and the electrostatic interactions is treated as default Amber parameters for GB simulation. The non-bond cutoff is 10 Å, and the time step is 1fs. Before we perform MD simulation, the entire system is first minimized for 3000 steps by the steepest descent method and then 6000 steps by the conjugate gradient method. Simulations at each temperature contain ten 10ns-trajectories with different initial atomic velocities. The total simulation time is up to 1.6 µs.

### Trajectory and Structure Analysis

We analyzed the trajectories using the Ptraj program in AMBER 11 for calculating the root mean square deviation (RMSD) as well as the native contacts. All the RMSD values are calculated against the experimental structures by using all heavy atoms. We employ the fraction of native contacts (NC for short below) as a measure of secondary and tertiary structure formation during unfolding process. We define the native contact using the distance cutoff 7 Å and calculate the fraction of native contacts by averaging all ten simulation trajectories. Such representation of native contact contains the base-pairing interactions as well as base stacking between neighboring bases. We give the corresponding smoothed curves of native contacts to better visualize their evolutions. The free energy landscape is calculated using the algorithm proposed by Pande et al. [16], in which the free energy is defined as 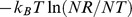, where *NR* is the number of structures in each region and *NT* is the total number of the structures. Although our simulated data are taken from the non-equilibrium unfolding trajectories, the free energy landscape also provides us the mostly populated structures, the unfolding intermediates, and possible unfolding pathway. The trajectory visualization and figures are generated using VMD [33], UCSF Chimera packages [34] and the PyMOL Molecular Graphics System, Version 1.3, Schrödinger, LLC.

To determine the unfolding temperature of our simulation system, we first did short thermodynamic simulations in which the aptamer was simulated over a wide temperature range (280 K to 440 K) to capture the RNA unfolding transition. The heat capacity *C_V_* is calculated to monitor aptamer folding and unfolding, following the equation *C_V_* = σ^2^_E_/*k*_B_ *T* ^2^, where σ_E_ is the energy fluctuation at a given temperature *T* and *k_B_* is the Boltzmann constant. We found that the melting temperature for Bsu preQ_1_ riboswitch is 380 K in both presence and absence of the ligand. The Tte preQ_1_ riboswitch has a little higher melting temperature in both states (390 K), which indicates that the Tte preQ_1_ riboswitch is more stable during the unfolding progress (see Figures S5 and S6). Therefore, we mainly focus on the simulations of unfolding processes at 400 K, which is slightly higher than the melting temperature and enables the RNA molecules unfold within a practically feasible time.

### Conclusion

In summary, we have performed 400 K all-atom unfolding molecular dynamics simulations of the unfolding of Bsu and Tte preQ_1_ riboswitch aptamers with and without ligands. The results indicate that the global behaviors of their unfolding are very similar and along a single directional pathway from the 3′ to 5′ end. Our results also show that there exist two intermediate states during the unfolding progress in the presence of ligand. In the presence of ligand the experimental ligand-bound structures are their stable states and also the functional “OFF” states. In the absence of ligands their stable states are not the experimental ligand-free structures (or the ligand-bound one by moving the ligand) but just one of the intermediate states in the presence of ligands. These ligand-free stable states seem to be the functional “ON” state. Our unfolding studies suggest that the regulation mechanisms of Bsu and Tte preQ_1_ riboswitch may be similar: the aptamers will form the functional “OFF” or “ON” states depending on ligand binding or not after the formation of the triplex P1-L3. Although the ligand-free state of the Tte preQ_1_ riboswitch aptamer has the same structure as its ligand-bound state, it also regulates the translational process through kinetic control as the Bsu preQ_1_ riboswitch. These results will also help the understanding of regulation mechanism of different preQ_1_ riboswitches as well as other types of riboswitches.

## Supporting Information

Figure S1 Dynamics of the two types of preQ1 riboswitch aptamer domain with (top) and without (bottom) ligand at 300 K. The different curves describe the time evolution of the average fraction of native contacts of the aptamer and its various segments during the unfolding simulations.
(TIF)

Figure S2 The two-dimensional free energy landscape of the two types of preQ1 aptamer domain with (left) and without (right) the ligand at 300 K. The order parameters are the fractions of native contacts for P1-L3 and P2-L1-L2, respectively.
(TIF)

Figure S3 The variation of RMSD as well as the distance among nucleotides in pseudoknot of the two types of preQ1 aptamer domain with (left) and without (right) the ligand at 300 K.
(TIF)

Figure S4 The variation of RMSD as well as the distance among nucleotides in pseudoknot of the two types of preQ1 aptamer domain with (left) and without (right) the ligand at 400 K. Only the first 2 ns are plotted in order to view more clearly.
(TIF)

Figure S5 Heat capacity profile as a function of temperature for the preQ1 riboswitch aptamer domain with (solid) and without (dot) preQ1.
(TIF)

Figure S6 Average fraction of native contacts (blue) and rmsd (red) of RNA as a function of temperatures in two different states. Standard deviation of the average values at each temperature are shown as error bars. (a) in the presence of ligand. (b) in the absence of ligand.
(TIF)

## References

[1] Breaker RR (2012) Riboswitches and the RNA world. Cold Spring Harb Perspect Biol 4. 10.1101/cshperspect.a003566 PMC3281570 21106649

[2] Barrick JE, Breaker RR (2007) The distributions, mechanisms, and structures of metabolite-binding riboswitches. Genome Biol 8: R239. 17997835 10.1186/gb-2007-8-11-r239 PMC2258182

[3] Serganov A, Patel DJ (2007) Ribozymes, riboswitches and beyond: regulation of gene expression without proteins. Nat Rev Genet 8: 776–790. 17846637 10.1038/nrg2172 PMC4689321

[4] Serganov A (2010) Determination of riboswitch structures: light at the end of the tunnel? RNA Biol 7: 98–103. 20061809 10.4161/rna.7.1.10756

[5] Stoddard CD, Montange RK, Hennelly SP, Rambo RP, Sanbonmatsu KY, et al (2010) Free state conformational sampling of the SAM-I riboswitch aptamer domain. Structure 18: 787–797. 20637415 10.1016/j.str.2010.04.006 PMC2917978

[6] Batey RT, Gilbert SD, Montange RK (2004) Structure of a natural guanine-responsive riboswitch complexed with the metabolite hypoxanthine. Nature 432: 411–415. 15549109 10.1038/nature03037

[7] Liberman JA, Wedekind JE (2011) Riboswitch structure in the ligand-free state. Wiley Interdiscip Rev RNA. 10.1002/wrna.114 PMC3252462 21957061

[8] Haller A, Souliere MF, Micura R (2011) The Dynamic Nature of RNA as Key to Understanding Riboswitch Mechanisms. Acc Chem Res. 10.1021/ar200035g 21678902

[9] Kang M, Peterson R, Feigon J (2009) Structural Insights into riboswitch control of the biosynthesis of queuosine, a modified nucleotide found in the anticodon of tRNA. Mol Cell 33: 784–790. 19285444 10.1016/j.molcel.2009.02.019

[10] Rieder U, Kreutz C, Micura R (2010) Folding of a transcriptionally acting preQ1 riboswitch. Proc Natl Acad Sci U S A 107: 10804–10809. 20534493 10.1073/pnas.0914925107 PMC2890745

[11] Jenkins JL, Krucinska J, McCarty RM, Bandarian V, Wedekind JE (2011) Comparison of a preQ1 riboswitch aptamer in metabolite-bound and free states with implications for gene regulation. J Biol Chem 286: 24626–24637. 21592962 10.1074/jbc.M111.230375 PMC3137038

[12] Ferre-D’Amare AR, Klein DJ, Edwards TE (2009) Cocrystal structure of a class I preQ(1) riboswitch reveals a pseudoknot recognizing an essential hypermodified nucleobase. Nature Structural & Molecular Biology 16: 343–344. 10.1038/nsmb.1563 PMC2657927 19234468

[13] Feng J, Walter NG, Brooks CL, 3rd (2011) Cooperative and directional folding of the preQ1 riboswitch aptamer domain. J Am Chem Soc 133: 4196–4199. 21375305 10.1021/ja110411m PMC3109358

[14] Lazaridis T, Karplus M (1997) “New view” of protein folding reconciled with the old through multiple unfolding simulations. Science 278: 1928–1931. 9395391 10.1126/science.278.5345.1928

[15] Brooks CL, 3rd (1998) Simulations of protein folding and unfolding. Curr Opin Struct Biol 8: 222–226. 9631297 10.1016/s0959-440x(98)80043-2

[16] Pande VS, Rokhsar DS (1999) Molecular dynamics simulations of unfolding and refolding of a beta-hairpin fragment of protein G. Proc Natl Acad Sci U S A. 96: 9062–9067. 10.1073/pnas.96.16.9062 PMC17732 10430895

[17] Daggett V, Levitt M (1993) Protein unfolding pathways explored through molecular dynamics simulations. J Mol Biol 232: 600–619. 7688428 10.1006/jmbi.1993.1414

[18] Dinner AR, Karplus M (1999) Is protein unfolding the reverse of protein folding? A lattice simulation analysis. Journal of Molecular Biology 292: 403–419. 10493884 10.1006/jmbi.1999.3051

[19] Sorin EJ, Engelhardt MA, Herschlag D, Pande VS (2002) RNA simulations: probing hairpin unfolding and the dynamics of a GNRA tetraloop. J Mol Biol 317: 493–506. 11955005 10.1006/jmbi.2002.5447

[20] Sarkar K, Nguyen DA, Gruebele M (2010) Loop and stem dynamics during RNA hairpin folding and unfolding. RNA 16: 2427–2434. 20962040 10.1261/rna.2253310 PMC2995403

[21] Csaszar K, Spackova N, Stefl R, Sponer J, Leontis NB (2001) Molecular dynamics of the frame-shifting pseudoknot from beet western yellows virus: the role of non-Watson-Crick base-pairing, ordered hydration, cation binding and base mutations on stability and unfolding. J Mol Biol 313: 1073–1091. 11700064 10.1006/jmbi.2001.5100

[22] Zhang Y, Zhang J, Wang W (2011) Atomistic analysis of pseudoknotted RNA unfolding. J Am Chem Soc 133: 6882–6885. 21500824 10.1021/ja1109425

[23] Zhang Q, Kang M, Peterson RD, Feigon J (2011) Comparison of solution and crystal structures of preQ1 riboswitch reveals calcium-induced changes in conformation and dynamics. J Am Chem Soc 133: 5190–5193. 21410253 10.1021/ja111769g PMC3085290

[24] Nissen P, Ippolito JA, Ban N, Moore PB, Steitz TA (2001) RNA tertiary interactions in the large ribosomal subunit: the A-minor motif. Proc Natl Acad Sci U S A 98: 4899–4903. 11296253 10.1073/pnas.081082398 PMC33135

[25] D.A. Case TAD, T.E Cheatham, III, C.L Simmerling, J Wang, R.E Duke, R Luo, R.C Walker, W Zhang, K.M Merz, B Roberts, B Wang, S Hayik, A Roitberg, G Seabra, I Kolossvary, K.F Wong, F Paesani, J Vanicek, X Wu, S.R Brozell, T Steinbrecher, H Gohlke, Q Cai, X Ye, J Wang, M. J Hsieh, G Cui, D.R Roe, D.H Mathews, M.G Seetin, C Sagui, V Babin, T Luchko, S Gusarov, A Kovalenko, and P.A Kollman (2010) AMBER 11. University of California, San Francisco.

[26] Gong Z, Xiao Y (2010) RNA stability under different combinations of amber force fields and solvation models. J Biomol Struct Dyn 28: 431–441. 20919758 10.1080/07391102.2010.10507372

[27] Zhao Y, Gong Z, Xiao Y (2011) Improvements of the hierarchical approach for predicting RNA tertiary structure. J Biomol Struct Dyn 28: 815–826. 21294592 10.1080/07391102.2011.10508609

[28] Banas P, Hollas D, Zgarbova M, Jurecka P, Orozco M, et al (2010) Performance of Molecular Mechanics Force Fields for RNA Simulations: Stability of UUCG and GNRA Hairpins. Journal of Chemical Theory and Computation 6: 3836–3849. 10.1021/ct100481h PMC8916691 35283696

[29] Yildirim I, Stern HA, Tubbs JD, Kennedy SD, Turner DH (2011) Benchmarking AMBER force fields for RNA: comparisons to NMR spectra for single-stranded r(GACC) are improved by revised chi torsions. J Phys Chem B 115: 9261–9270. 21721539 10.1021/jp2016006 PMC3140773

[30] Zgarbova M, Otyepka M, Sponer J, Mladek A, Banas P, et al (2011) Refinement of the Cornell, et al. Nucleic Acids Force Field Based on Reference Quantum Chemical Calculations of Glycosidic Torsion Profiles. J Chem Theory Comput 7: 2886–2902. 21921995 10.1021/ct200162x PMC3171997

[31] Tsui V, Case DA (2000) Theory and applications of the generalized Born solvation model in macromolecular Simulations. Biopolymers 56: 275–291. 11754341 10.1002/1097-0282(2000)56:4<275::AID-BIP10024>3.0.CO;2-E

[32] Loncharich RJ, Brooks BR, Pastor RW (1992) Langevin dynamics of peptides: the frictional dependence of isomerization rates of N-acetylalanyl-N’-methylamide. Biopolymers 32: 523–535. 1515543 10.1002/bip.360320508

[33] Humphrey W, Dalke A, Schulten K (1996) VMD: visual molecular dynamics. J Mol Graph 14: 33–38, 27–38. 10.1016/0263-7855(96)00018-5 8744570

[34] Pettersen EF, Goddard TD, Huang CC, Couch GS, Greenblatt DM, et al (2004) UCSF Chimera–a visualization system for exploratory research and analysis. J Comput Chem 25: 1605–1612. 15264254 10.1002/jcc.20084

