# Supplementary figures and images for: Computational Study of Unfolding and Regulation Mechanism of preQ1 Riboswitches
Source: PLoS One. 2012 Sep 17;7(9):e45239. doi: 10.1371/journal.pone.0045239 (PMC3444477; doi:10.1371/journal.pone.0045239)

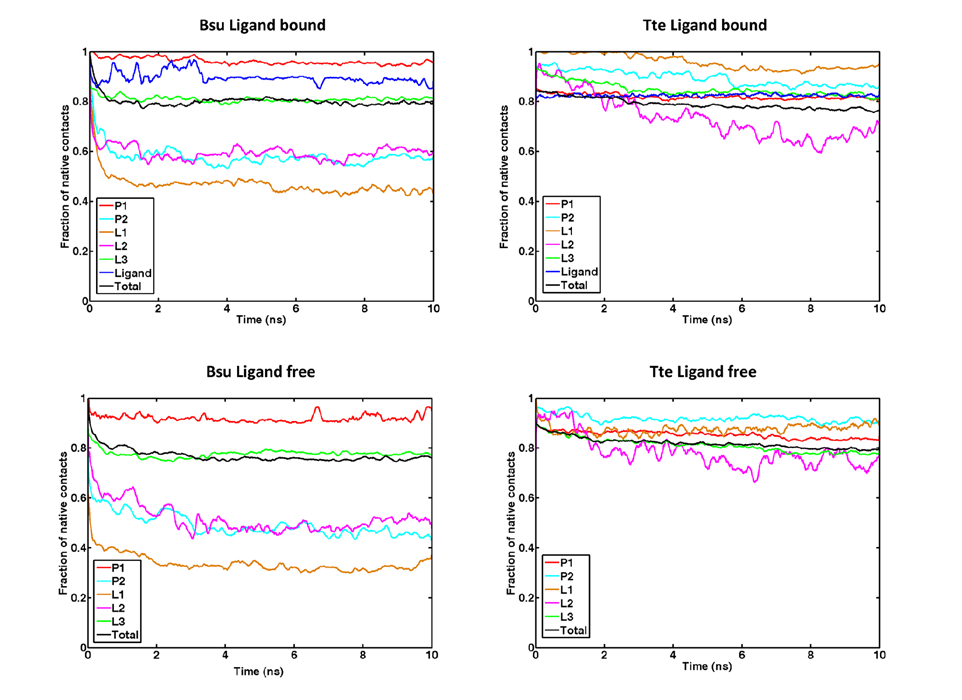

Supplement: Figure S1 — Dynamics of the two types of preQ1 riboswitch aptamer domain with (top) and without (bottom) ligand at 300 K. The different curves describe the time evolution of the average fraction of native contacts of the aptamer and its various segments during the unfolding simulations. (TIF) [file pone.0045239.s001.tif]

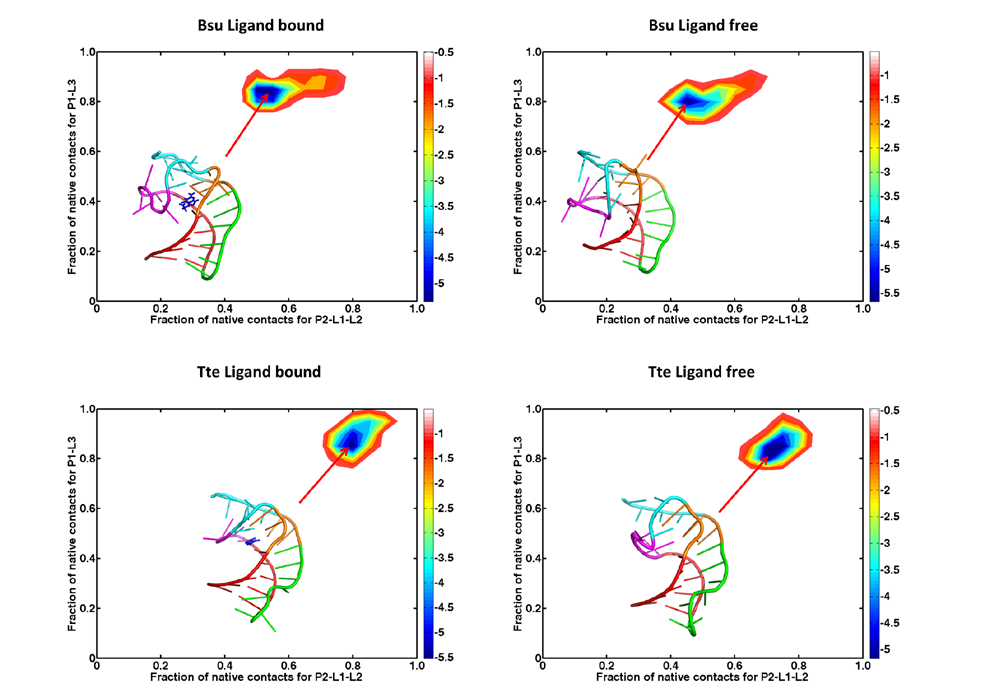

Supplement: Figure S2 — The two-dimensional free energy landscape of the two types of preQ1 aptamer domain with (left) and without (right) the ligand at 300 K. The order parameters are the fractions of native contacts for P1-L3 and P2-L1-L2, respectively. (TIF) [file pone.0045239.s002.tif]

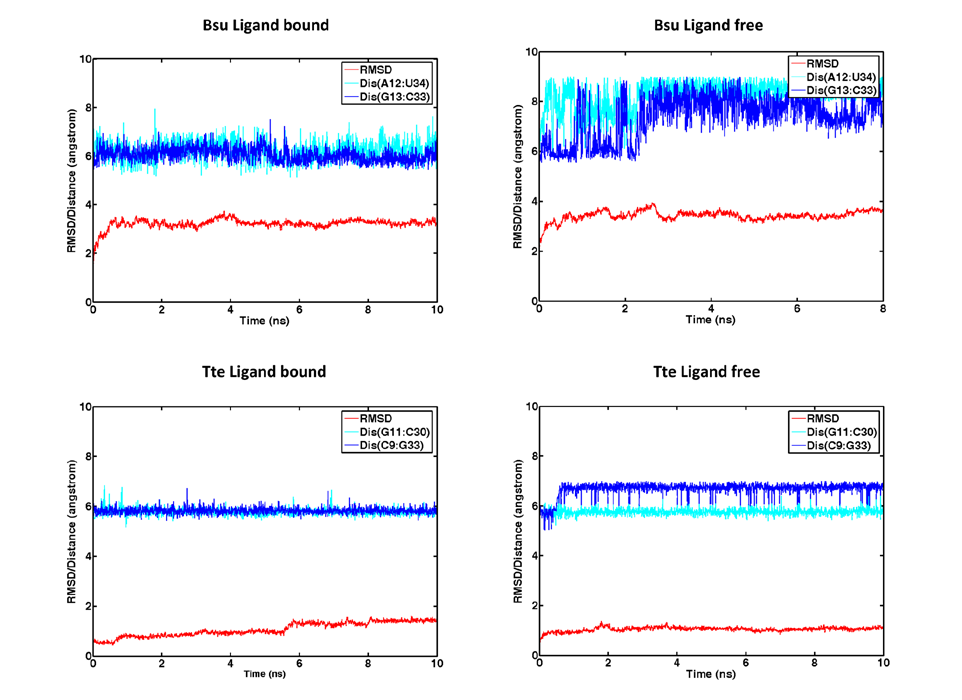

Supplement: Figure S3 — The variation of RMSD as well as the distance among nucleotides in pseudoknot of the two types of preQ1 aptamer domain with (left) and without (right) the ligand at 300 K. (TIF) [file pone.0045239.s003.tif]

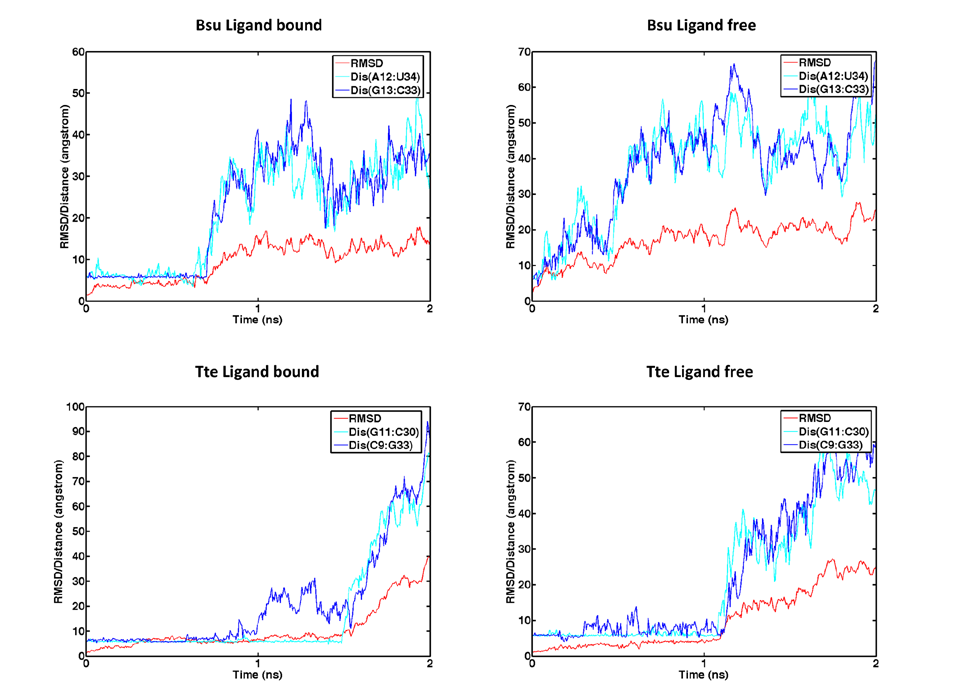

Supplement: Figure S4 — The variation of RMSD as well as the distance among nucleotides in pseudoknot of the two types of preQ1 aptamer domain with (left) and without (right) the ligand at 400 K. Only the first 2 ns are plotted in order to view more clearly. (TIF) [file pone.0045239.s004.tif]

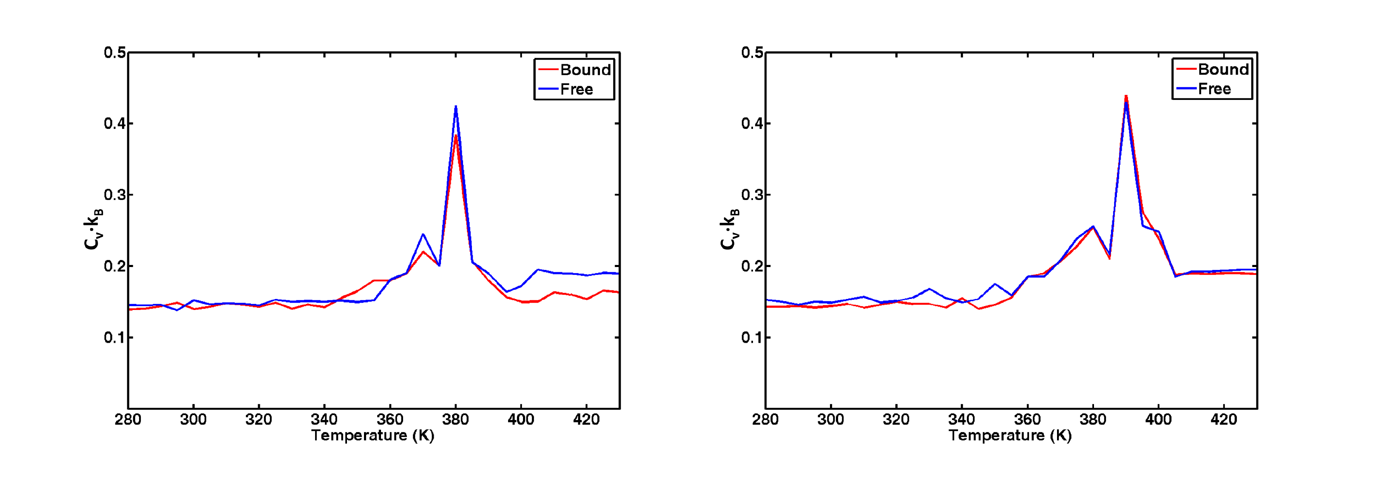

Supplement: Figure S5 — Heat capacity profile as a function of temperature for the preQ1 riboswitch aptamer domain with (solid) and without (dot) preQ1. (TIF) [file pone.0045239.s005.tif]

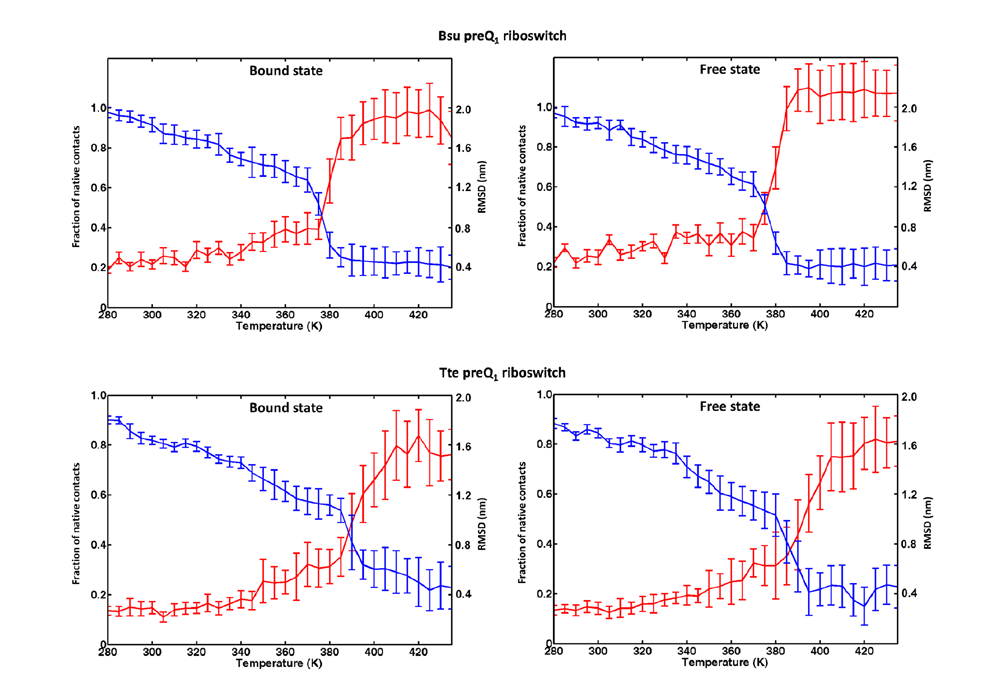

Supplement: Figure S6 — Average fraction of native contacts (blue) and rmsd (red) of RNA as a function of temperatures in two different states. Standard deviation of the average values at each temperature are shown as error bars. (a) in the presence of ligand. (b) in the absence of ligand. (TIF) [file pone.0045239.s006.tif]
